# Wing Planform Effect on the Aerodynamics of Insect Wings

**DOI:** 10.3390/insects13050459

**Published:** 2022-05-13

**Authors:** Hao Li, Mostafa R. A. Nabawy

**Affiliations:** 1 Department of Mechanical, Aerospace and Civil Engineering, The University of Manchester, Manchester M1 3BB, UK; hao.li@manchester.ac.uk; 2 Aerospace Engineering Department, Faculty of Engineering, Cairo University, Giza 12613, Egypt

**Keywords:** insect flight, flapping wings, aerodynamics, wing planform, leading-edge vortex

## Abstract

**Simple Summary:**

This study aims to provide an improved understanding of the effect of wing planform shape on the aerodynamic performance of insect flapping wings. We focus our investigation on three planform parameters, namely aspect ratio, radial centroid location, and wing root offset, and their effect on the aerodynamic performance is characterised at a flow Reynolds number most relevant to small insects similar to fruit flies. We show that aspect ratio and root offset mainly influence the flow detachment area near the wingtip, whereas radial centroid location mainly influences the local flow evolution time on the wing surface. Overall, increasing the aspect ratio is beneficial to lift and efficiency up to a limit where flow detachment near the wing tip leads to less-favorable performance. Similarly, increasing the wing root offset leads to an increased flow detachment area near the wing tip, resulting in reduced lift coefficient, but the aerodynamic efficiency remains relatively unaffected by the root offset value for most aspect ratios. Finally, increasing the radial centroid location mainly increases the aerodynamic efficiency.

**Abstract:**

This study investigates the effect of wing planform shape on the aerodynamic performance of insect wings by numerically solving the incompressible Navier-Stokes equations. We define the wing planforms using a beta-function distribution and employ kinematics representative of normal hovering flight. In particular, we use three primary parameters to describe the planform geometry: aspect ratio, radial centroid location, and wing root offset. The force coefficients, flow structures, and aerodynamic efficiency for different wing planforms at a Reynolds number of 100 are evaluated. It is found that the wing with the lowest aspect ratio of 1.5 results in the highest peaks of lift and drag coefficients during stroke reversals, whereas the higher aspect ratio wings produce higher lift and drag coefficients during mid half-stroke translation. For the wings considered, the leading-edge vortex detachment is found to be approximately at a location that is 3.5–5 mean chord lengths from the wing center of rotation for all aspect ratios and root offsets investigated. Consequently, the detachment area increases with the increase of aspect ratio and root offset, resulting in reduced aerodynamic coefficients. The radial centroid location is found to influence the local flow evolution time, and this results in earlier formation/detachment of the leading-edge vortex for wings with a smaller radial centroid location. Overall, the best performance, when considering both average lift coefficient and efficiency, is found at the intermediate aspect ratios of 4.5–6; increasing the centroid location mainly increases efficiency; and increasing the root offset leads to a decreased average lift coefficient whilst leading to relatively small variations in aerodynamic efficiency for most aspect ratios.

## 1. Introduction

Flying insects are prevalent in nature and offer diverse examples of successful flight concepts. Despite their prolonged existence, serious steps towards understanding how insects fly only started in the past few decades. In fact, our understanding of insect wing aerodynamics has lately been significantly improved, thanks to the numerous studies that considered the primary aerodynamic mechanisms enabling insects to stay aloft while hovering. These mechanisms include: (1) the formation of a stably attached leading-edge vortex (LEV), which allows reattachment of flow on the wing’s upper surface, preventing stall and augmenting lift production [1,2,3,4]; (2) rotational circulation due to the wing pitching rotation at the end of each half-stroke, which is a main contributor to the transient force production during stroke reversals [5]; (3) added mass effects, resulting from the periodic acceleration and deceleration of the flapping wing, again influencing transient force production mainly around stroke reversals [6]; and (4) wake capture, which is a unique aerodynamic mechanism for flapping wings, arising from the interaction of the wing with wake shed from the preceding half-stroke [1,5,7]. Despite the current progress, there is still limited understanding of how the aerodynamic behaviour of flapping insect wings changes for different wing morphologies. The objective of this paper is, therefore, to provide an improved understanding of the physics underlying insect-like flapping wing aerodynamics through identifying the key morphological variables that can enhance aerodynamic performance at this scale.

Early studies concerned with wing planform shape effects primarily focused on aerodynamic force production. Usherwood and Ellington [8] conducted an experimental campaign using revolving wing models of hawkmoth-like planform shape to study the effect of aspect ratio on aerodynamic force production. By changing the wing aspect ratio within a range between 2.27 and 7.92, they found that, when the wing angle of attack was less than 65°, aspect ratio had little effect on aerodynamic force coefficients; nevertheless, higher aspect ratio wings still resulted in a steeper growth in lift coefficient with the increase of angle of attack. Luo and Sun [9] used Computational Fluid Dynamics (CFD) simulations to study the aerodynamic force production of different wing planform shapes obtained from 10 different insect species. Their results showed that wing planform had minor influence on the aerodynamic force coefficients. However, they only considered a small range of aspect ratio values, between 2.84 and 5.45. Shahzad et al. [10] also used CFD simulations to study the effect of radial centroid location and aspect ratio on the aerodynamic performance of hovering insect-like flapping wings. The planform shapes in their study were defined using the beta-function distribution, proposed by Ellington [11] as representative of insect wing planform shapes. Their study investigated wings with aspect ratio and radial centroid location values varying between 1.5–6.0 and 0.43–0.63, respectively. They found that lift coefficient increased with the increase of aspect ratio, whereas aerodynamic efficiency defined as the ratio of average lift coefficient to power coefficient reached its maximum value at an aspect ratio of 2.96. On the other hand, increasing the radial centroid location was found to decrease the hovering efficiency. However, in their study, a sinusoidal waveform was employed for both flapping and pitching kinematics. Hence, the contribution from wing rotational/acceleration effects during stroke reversals was rather weak, despite the fact that these effects can have a significant influence on insect wing aerodynamic performance [5].

Several analytical studies have also been conducted to better understand the wing planform effects within revolving/flapping motions. Ansari et al. [12] developed a potential flow-based model and used it to assess the effect of different wing planform shapes. They found an increasing trend of lift with aspect ratio and root offset. They, additionally, proposed that more area at the wing outboard region is beneficial for lift force production, due to the higher velocity towards the wing tip. Nabawy and Crowther [13] developed a quasi-steady lifting line theory and used it to investigate the aerodynamic performance of revolving/flapping wings experiencing fully attached flows. They showed that increasing the aspect ratio and decreasing the wing radial centroid location increases the wing lift coefficient. In a series of studies using analytical models [14,15,16], Nabawy and Crowther identified the optimum wing planform shape that would minimize the induced power factor, a metric for lift efficiency production. They showed that as more wing area is allocated near the wing root (smaller radial centroid location), the more efficient the planform becomes with respect to induced power effects. Moreover, they identified the elliptic wing planform as the optimum planform when profile power effects are concerned. In a study on the effect of wing morphology on aerodynamics of wings in flies, Krishna et al. [17] used numerical simulation to compare the aerodynamic force and power of a so-called ‘ideal-shaped’ wing, a rectangular wing, and a wing planform similar to that of a blowfly, all with the same wing area and span. The ‘ideal-shaped’ wing planform was derived analytically based on the optimal distribution of circulation along the wingspan, taken from Prandtl and Betz [18,19]. They found that for both revolving and flapping kinematics the rectangular wing and fly wing showed similar lift force and power performance, whereas the ‘ideal-shaped’ wing, surprisingly, under-performed with respect to both lift and efficiency.

The effect of wing planform shape on the evolution and attachment of the LEV on insect wings has also been investigated. Phillips et al. [20,21] used a robotic flapping wing apparatus to experimentally study rectangular wings with different aspect ratios and root offsets at a Reynolds number of 1400. They found that for wing aspect ratios greater than three, the LEV starts to detach at approximately 70% of wingspan at mid half-stroke; furthermore, a higher aspect ratio results in earlier detachment of LEV. Moreover, they found that circulatory lift from the LEV peaks at an aspect ratio of six. Similar observations on the LEV detachment were found by Han et al. [22] for insect-like flapping wings with aspect ratios greater than three, thus proposing that the lift coefficient peaks at an aspect ratio of three. Harbig et al. [23] investigated *Drosophila* shaped wing planforms with aspect ratios between 2.9 and 7.3 using CFD simulations. Small differences in lift coefficient were found for wing aspect ratios between 2.9 and 5.1, but a decrease in lift coefficient was found when the aspect ratio was further increased to 7.3. This led them to propose that the reduction in LEV circulation near the wingtip at a high aspect ratio leads to the reduction in lift coefficient. Kruyt et al. [24] used particle image velocimetry (PIV) to study the LEV structures of revolving rectangular wings with different aspect ratios. They found that the LEV remains attached up to a spanwise location that is four times of the wing chord length, measured from the centre of rotation. Further outboard, the LEV lifts away from the wing surface, leading to tip stall and reduced aerodynamic efficiency.

Most recently, the effect of wing planform shape on LEV structure and aerodynamic performance has been investigated in connection to the Rossby number [25], defined as the ratio of the radius of gyration to the mean chord length. Such investigations are mainly useful for ascertaining the effect of the wing root offset (how distant the wing root station is from the centre of rotation) on aerodynamic performance. Lee et al. [26] studied rectangular revolving wings using CFD, with wing aspect ratios varying between 1 and 10. By changing the wing root offset, they forced the Rossby number to vary within a range between 0.58 and 7.57. They found that when the Rossby number is kept fixed, lift coefficient increases with the increase of aspect ratio until plateauing at aspect ratios between four and five. On the other hand, they showed that increasing Rossby number, for a given aspect ratio, leads to decreased lift coefficients due to the reduced stability of the LEV. Jardin and Colonius [27] also investigated rectangular revolving wings with aspect ratios ranging between 1 and 7, and Rossby numbers ranging between 1.73 and 5.77. They found that for fixed Rossby number cases, lift coefficient increases monotonically with aspect ratio, and that the increase rate is high for small aspect ratios but asymptotic for large aspect ratios. On the other hand, for fixed aspect ratio cases, lift coefficient decreases monotonically with the increase of the Rossby number. Similarly, Bhat et al. [28] also demonstrated a decreasing lift coefficient with the increase of the Rossby number in a numerical study of revolving wings. In fact, they proposed that the weakening of the LEV due to the reduced spanwise flow caused the reduction of lift at higher Rossby numbers.

Despite the abundance of results in the literature, arguably, our understanding of the effect of wing planform shape on the aerodynamics of insect wings is still limited. This is because many of the existing studies have focused mainly on the effect of aspect ratio, but provided less attention to investigating the radial centroid location and root offset effects, which are arguably prominent features in defining the wing planform shape. In fact, most of the investigated wings were either rectangular wings or of a specific insect-inspired shape; hence, a systematic variation of the morphological parameters for representative insect planforms is needed. Furthermore, most of the existing studies employed revolving wings for the simplicity they bring while investigating the flow structure and aerodynamic forces; however, insect wings employ reciprocating motions, where unsteady aerodynamic effects can be significant due to the cyclic formation of vortical structures. As such, in this study, we simultaneously investigate the effect of aspect ratio, radial centroid location, and root offset of insect wings to provide a detailed understanding of the holistic effect of planform shape on their aerodynamics performance. The wing geometry is defined using a beta-function distribution, known to provide accurate representation (within 5% error) of many natural insect wing planforms [11]. The kinematic functions used in this study provide fair representation of ‘normal hovering’ flight. The flow structures and aerodynamic force coefficients at a Reynolds number of 100, relevant to insects similar to fruit flies, are obtained by numerically solving the incompressible Navier-Stokes equations using the open-source CFD package OpenFOAM (Open-source Field Operation and Manipulation).

The rest of this paper is organised as follows: Section 2 provides the definition of the wing planform shapes and kinematic waveforms chosen for investigation. The setup and validation of the numerical model are also presented. Section 3 demonstrates the simulation results for different wing planform cases, including the influence of aspect ratio, radial centroid location, and root offset on the flow structure evolution, instantaneous and average aerodynamic force production, and power efficiency. Finally, Section 4 presents the conclusions of this study.

## 2. Materials and Methods

### 2.1. Wing Planforms and Kinematics

The wing planform representation used in this study is based on the beta-function distribution of wing area, conceived by Ellington [11]. Here, the non-dimensional wing chord length (i.e., local wing chord length divide by the mean chord, $\hat{c}\left(\hat{r}\right)=c\left(\hat{r}\right)/\bar{c}$) along the wing is given by [11]:

(1) $$\hat{c}\left(\hat{r}\right)=\frac{{\hat{r}}^{p-1}{\left(1-\hat{r}\right)}^{q-1}}{\int_{0}^{1}{\hat{r}}^{p-1}{\left(1-\hat{r}\right)}^{q-1}d\hat{r}}$$

where $\hat{r}=r/R$ is the non-dimensional radial position along the wing length, R. The parameters p and q are defined using the non-dimensional radial centroid location and the non-dimensional radius of gyration location, ${\hat{r}}_{1}$ and ${\hat{r}}_{2}$, respectively, as follows [11]:

(2) $$p={\hat{r}}_{1}\left(\frac{{\hat{r}}_{1}\left(1-{\hat{r}}_{1}\right)}{{\hat{r}}_{2}^{2}-{\hat{r}}_{1}^{2}}-1\right)$$

(3) $$q=\left(1-{\hat{r}}_{1}\right)\left(\frac{{\hat{r}}_{1}\left(1-{\hat{r}}_{1}\right)}{{\hat{r}}_{2}^{2}-{\hat{r}}_{1}^{2}}-1\right)$$

The parameters ${\hat{r}}_{1}$ and ${\hat{r}}_{2}$ for the wing planform shape are linked via the relation ${\hat{r}}_{2}=0.929{\left[{\hat{r}}_{1}\right]}^{0.732}$ proposed by Ellington [11]. As such, ${\hat{r}}_{2}$ value can be evaluated based on a prescribed value of ${\hat{r}}_{1}$. The aspect ratio of the wing is defined as:

(4) $$AR=\frac{r_{T}-r_{R}}{\bar{c}}$$

where $r_{T}$ and $r_{R}$ are the radial positions of the wing tip and wing root, respectively. The non-dimensional root offset ${\hat{r}}_{R}$ is defined by:

(5) $${\hat{r}}_{R}=\frac{r_{R}}{\bar{c}}$$

where $r_{R}\;$ is the distance by which the wing root station is displaced/offset from the centre of rotation.

**Figure 1:**
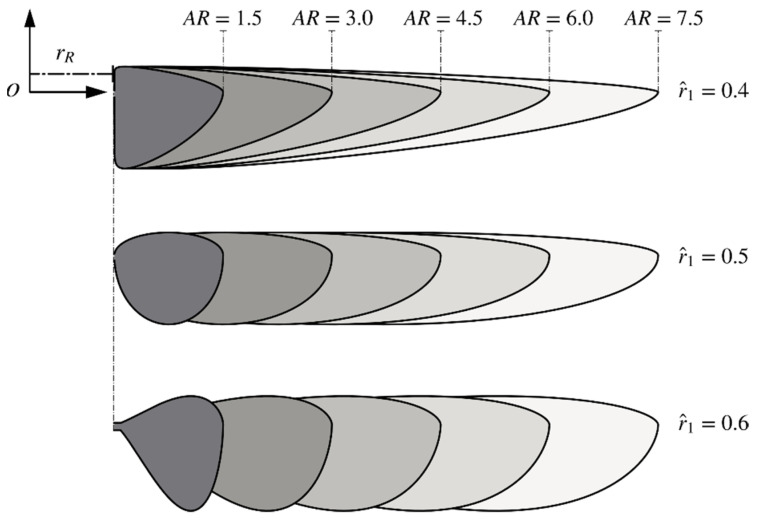
Wing planform shapes for different aspect ratios and non-dimensional radial centroid locations.

Based on the above definitions, the effect of wing planform shape on the aerodynamics of insect wings can be investigated by varying the aspect ratio, radial centroid location, and wing root offset. Note that other secondary planform parameters such as the shape of the leading-edge contour or the location of the pitching axis are not considered in this study. The aspect ratio range chosen for investigation is AR = 1.5–7.5, the radial centroid location is investigated within the range ${\hat{r}}_{1}$ = 0.4–0.6, and the non-dimensional wing root offset is investigated within the range ${\hat{r}}_{R}$ = 0–3. These chosen ranges for investigation are selected to ensure most insect wings planforms are covered, following data collected in [11]. The wing thickness is set as 5% of the mean chord length, $\bar{c}$, for all cases, and the axis of pitching rotation is constantly set at 0.25$\bar{c}$ from the leading-edge. The specific values considered in this study for aspect ratio, non-dimensional radial centroid location, and non-dimensional wing root offset are summarized in Table 1. The resulting wing planform morphologies for these geometric parameters are visualised in Figure 1.

The wing motion employed in this study is selected to represent lift-based ‘normal hovering’ kinematics of insect wings, where the flapping waveform is defined using a sinusoidal function near the beginning and end of the half-stroke, whereas between stroke reversals, the wing has constant velocity. The waveform function for flapping angular velocity at stroke reversal is, thus, given by [29]:

(6) $$\dot{\phi }\left(\hat{t}\right)=\pm {\dot{\phi }}_{M}\cos\left[\frac{\pi \left(\hat{t}-{\hat{t}}_{sa}\right)\;}{{\hat{t}}_{a}}\right],\;\;\;\;\;\;\;{\hat{t}}_{sa}\leq \hat{t}\leq {\hat{t}}_{sa}+{\hat{t}}_{a}$$

where ${\dot{\phi }}_{M}$ is the maximum flapping angular velocity; $\hat{t}=t/T$ is the non-dimensional time ranging between 0 and 1 within a flapping cycle; ${\hat{t}}_{a}=t_{a}/T$ ($t_{a}$ being the acceleration duration) is the total non-dimensional acceleration duration at the beginning and end of a half-stroke; and ${\hat{t}}_{sa}$ is the non-dimensional time at which wing acceleration starts.

**Figure 2:**
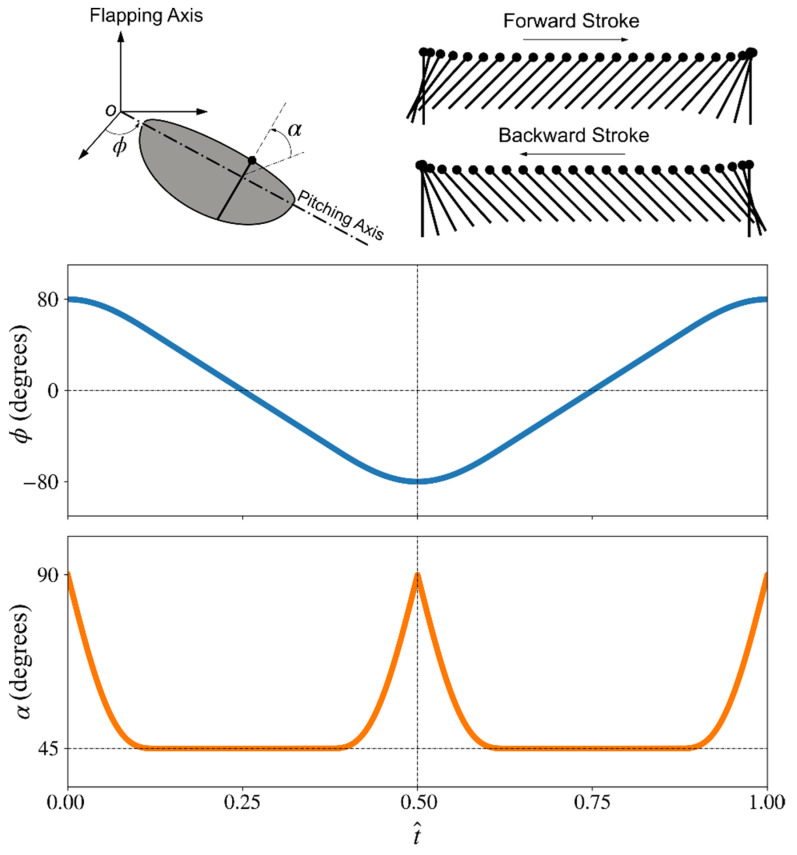
Employed wing kinematics waveforms for flapping and pitching.

The pitching kinematic waveform is defined similarly, using a sinusoidal function near the beginning and end of the half-stroke, while allowing a constant angle-of-attack between stroke reversals. The pitching angular velocity near stroke reversal is, thus, given by [29]:

(7) $$\dot{\theta }\left(\hat{t}\right)=\pm \frac{\Theta }{{\hat{t}}_{p}}\left\{1-\cos\left[\frac{2\pi \left(\hat{t}-{\hat{t}}_{sp}\right)}{{\hat{t}}_{p}}\right]\right\},\;\;{\hat{t}}_{sp}\leq \hat{t}\leq {\hat{t}}_{sp}+{\hat{t}}_{p}$$

where the wing pitch amplitude, Θ, is fixed as 90° for all wing planform shapes in the current study, corresponding to an angle of attack at mid half-stroke of 45°, known to allow maximum lift coefficient values [30]; ${\hat{t}}_{p}=t_{p}/T$ ($t_{p}$ being the wing pitch duration) is the total non-dimensional pitching duration during a half-stroke; and ${\hat{t}}_{sp}$ is the non-dimensional time at which wing pitching starts. In the current study, the flapping stroke amplitude is fixed as 160° for all wing planform shapes, which is close to the upper limit reported for insect wing kinematics [31]. The non-dimensional acceleration and pitching durations, ${\hat{t}}_{a}$ and ${\hat{t}}_{p}$, are chosen as 0.25. The wing kinematic waveforms for both flapping and pitching motions are shown in Figure 2. Note that, constant flapping angular velocity and constant angle of attack waveforms are known to be the most efficient waveforms in terms of aerodynamic power consumption to sustain a given amount of lift [30]. However, these waveforms are unrealistic for flapping flight realization near stroke reversals, despite being mathematically elegant. As such, the waveforms adopted here represent a good compromise that is close enough to these optimum waveforms while maintaining the practicality aspect.

The average velocity of the wing at radius of gyration, ${\hat{r}}_{2}$, over a flapping cycle, $U_{2}$, is used to define the Reynolds number for a given mean chord value and kinematic viscosity, ν, based on:

(8) $$Re=\frac{U_{2}\bar{c}}{\nu }$$

We only consider a Reynolds number of 100, which is relevant to small insects similar to fruit flies. It is worth noting here that the Reynolds number was found to have a relatively small influence on force production for insect-like flapping wings in several other studies [10,25,32,33]; however, some evidence showed that the mechanism for LEV stability (e.g., core-wise flow strength) is different between low and high Reynolds numbers [34].

In the current study, the flapping frequency for all planform cases is kept constant, and the kinematic viscosity is changed to ensure a constant Reynolds number value of 100 is achieved. This ensures that differences in aerodynamic and flow features observed from our simulations for the different wings considered are due to planform effects.

Finally, it should be noted that we model insect wings as rigid surfaces; hence, the effects of wing twist along the span, which is normally observed in real insects, is not considered. Given that the twist distribution contributes towards controlling the load distribution on a wing surface, it is not unexpected that any twist distribution resulting from wing flexibility may have an influence on the LEV development and detachment. Thus, this effect is an important direction to be further investigated in future studies. That said, it should be noted that a wide range of previous studies have also shown that rigid wings are able to capture the primary aerodynamic characteristics of hovering insect wings with reasonable accuracy. For example, Du and Sun [35] used numerical simulations to study the effects of camber and spanwise twist deformations for the wings of a hovering hoverfly (*Eristalis tenax*). Their model showed that the time courses of lift, drag, and aerodynamic power coefficients for a deformable wing are very similar to that produced by a rigid wing, although a 10% higher lift was found for the flexible wing. Using a fluid-structure interaction simulation, Nakata and Liu [36] analysed the aerodynamic performance for the hovering flexible wings of the hawkmoth (*Manduca sexta*). They found that wing flexibility can increase lift production but also costs more power. Overall, only a 3.4% increase in aerodynamic efficiency was found for the flexible wing when compared to a rigid wing. As a final example, Zhao et al. [37] used a dynamically-scaled mechanical model to experimentally measure the aerodynamic forces on flapping wings with variable trailing-edge flexural stiffness at a Reynolds number of 2000. They showed that for low to medium angles of attack, increasing flexibility decreases the aerodynamic force production but the lift-to-drag ratio remains approximately constant. It was also noted in their study that the instantaneous force traces reveal no major differences in the underlying modes of force generation between flexible and rigid wings.

### 2.2. Numerical Simulation

The current study solves the incompressible Navier-Stokes equations for the flow around the 3D wing:

(9) $$\frac{\partial u_{i}}{\partial x_{i}}=0$$

(10) $$\frac{\partial u_{i}}{\partial t}+\frac{\partial }{\partial x_{j}}\left(u_{j}u_{i}\right)=-\frac{1}{\rho }\frac{\partial p}{\partial x_{i}}+\nu \nabla^{2}u_{i}$$

where $u_{i}$ and $u_{j}$ denote the flow velocity components in the global Cartesian coordinate system; $x_{i}$ and $x_{j}$ denote the coordinate components within the domain; t denotes time; p denotes pressure; and ρ denotes the fluid density.

The open-source package OpenFOAM was employed to solve the above equations using a cell-centred finite volume method. For the incompressible flow, a segregated PIMPLE algorithm, which integrates a PISO (Pressure-Implicit with Splitting of Operators) inner loop and a SIMPLE (Semi-Implicit Method for Pressure Linked Equations) outer corrector loop, is used to solve the pressure-velocity coupling [38,39]. A second order central/backward differencing scheme is used for spatial gradient/time evolution terms. The pressure equation is solved using a GAMG (Geometric-algebraic Multi Grid) method, and the velocity equation is solved using a Gauss-Seidel method.

**Figure 3:**
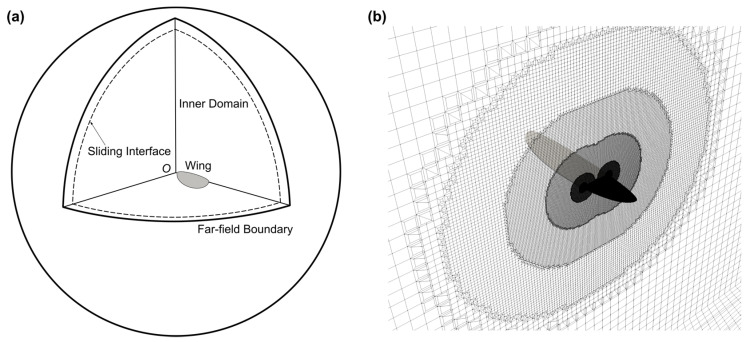
Computational domain and mesh setup. (**a**) Domain and boundary definitions; (**b**) mesh distribution near the wing surface; note that extra refinement was applied at both the wing’s leading- and trailing-edges.

The computational set-up consists of a stationary outer spherical domain and a moving inner spherical domain with the wing placed at the origin. The outer domain far-field boundary has a uniform fixed zero values for (gauge) pressure and velocity gradient. A no-slip boundary condition is applied at the wing surface. The inner and outer domains are linked with a spherical sliding interface. The inner sphere has a radius of 36$\bar{c}$ and the outer sphere has a radius of 40$\bar{c}$ for all the wing planforms, which ensures that the flow disturbances resulting from wing motions have negligible influence on the far-field boundary. The computational meshes for the different wing planforms are generated using the snappyHexMesh utility within OpenFOAM. The computational domain and mesh distribution near the wing surface are shown in Figure 3.

Throughout the simulations, the inner domain rotates about the origin to produce the required flapping and pitching motions. The inverse distance algorithm is used as a means for interpolating the fluid variables at the sliding interface. Thus, no mesh deformation or re-meshing is required, which ensures a high-quality mesh throughout the simulations.

The lift, drag, and power coefficients are evaluated using the relations:

(11) $$C_{L}\left(\hat{t}\right)=\frac{2L\left(\hat{t}\right)}{\rho U_{2}^{2}S}$$

(12) $$C_{D}\left(\hat{t}\right)=\frac{2D\left(\hat{t}\right)}{\rho U_{2}^{2}S}$$

(13) $$C_{P}\left(\hat{t}\right)=\frac{2P\left(\hat{t}\right)}{\rho U_{2}^{3}S}$$

where $L\left(\hat{t}\right)$ and $D\left(\hat{t}\right)$ are the instantaneous lift and drag forces; $P\left(\hat{t}\right)$ is the aerodynamic power at time instance $\hat{t}$, obtained from the inner product of aerodynamic moment, $\vec{M}\left(\hat{t}\right)$, and angular velocity, $\vec{\omega }\left(\hat{t}\right)$:

(14) $$P\left(\hat{t}\right)=\vec{M}\left(\hat{t}\right)\cdot \vec{\omega }\left(\hat{t}\right)$$

The aerodynamic performance is also assessed using the average lift, drag, and aerodynamic power coefficients over a flapping cycle, based on the relations:

(15) $${\bar{C}}_{L}=\int_{0}^{1}C_{L}\left(\hat{t}\right)d\hat{t}$$

(16) $${\bar{C}}_{D}=\int_{0}^{1}C_{D}\left(\hat{t}\right)d\hat{t}$$

(17) $${\bar{C}}_{P}=\int_{0}^{1}C_{P}\left(\hat{t}\right)d\hat{t}$$

The mass specific power, $P^{*}$, [40] is expressed as:

(18) $$P^{*}=\frac{\int_{0}^{1}P\left(\hat{t}\right)d\hat{t}}{Mg}$$

where M is the total mass, and g is the gravitational acceleration. Assuming lift equals to weight, $\int_{0}^{1}L\left(\hat{t}\right)d\hat{t}=Mg$, which reasonably holds for insects in hovering flight. The above equation reduces to:

(19) $$P^{*}=U_{2}\times \frac{{\bar{C}}_{P}}{{\bar{C}}_{L}}=\sqrt{\frac{2Mg}{\rho {\bar{C}}_{L}S}}\times \frac{{\bar{C}}_{P}}{{\bar{C}}_{L}}\;=\sqrt{\frac{2Mg}{\rho S}}\times \frac{{\bar{C}}_{P}}{{\bar{C}}_{L}^{3/2}}$$

In the above equation, the first term is constant for a given mass and wing area; hence, the second term can be used as a metric for evaluating aerodynamic efficiency. In the current study, the inverse of this second term, which we name as power factor, $P_{f}$, due to the similarity with the classical power factor for fixed wing motions ($C_{L}^{3/2}/C_{D}$) [25,41], is defined as:

(20) $$P_{f}=\frac{{\bar{C}}_{L}^{3/2}}{{\bar{C}}_{P}}$$

The above definition of the power factor is used in the current study to measure the aerodynamic efficiency for different wing planform shapes.

In the current study, the vortical structures are identified using the well-known Q-criterion [42]. It is worth mentioning, at this stage, that the simulations in this study are conducted using the Computational Shared Facility (CSF3) at the University of Manchester comprising a range of CPU cores from Intel Xeon E5-2640 Sandy Bridge to Intel Xeon Gold 6130 Broadwell. Each simulation case is set using 32 cores running in parallel. The computational time for each case is between 48 to 72 h, depending on the wing planform shape.

### 2.3. Convergence Assessment

**Figure 4:**
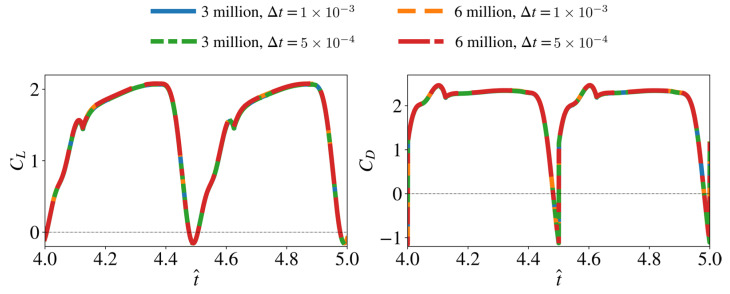
Mesh and time step convergence results. Negligible difference between cases is evident.

For our simulations, mesh systems with approximately 2.0–5.6 million cells are employed, which have approximately 29,000–144,000 nodes on the wing surface, depending on the wing planform case. The time step size (Δt) is set as 1 × 10^−3^ (relative to the flapping cycle, T). These numerical settings are decided based on a convergence study that compared the results obtained from the mesh with three million cells and a time step size of 1 × 10^−3^ to a higher-density mesh with six million cells and a smaller time step size of 5 × 10^−4^ for a baseline wing geometry with AR = 3, ${\hat{r}}_{1}$ = 0.5, ${\hat{r}}_{R}$ = 0, representing a wing geometry typically found in insects [11,25]. The comparison of the instantaneous lift and drag coefficients from this convergence study is presented in Figure 4. The average force coefficients are also provided in Table 2. The results show that the maximum difference in ${\bar{C}}_{L}$ and ${\bar{C}}_{D}$ for the different mesh and time step size cases is less than 0.7%. As such, the mesh size with three million cells and the time step size of 1 × 10^−3^ are deemed acceptable for the baseline wing geometry demonstrated here.

**Figure 5:**
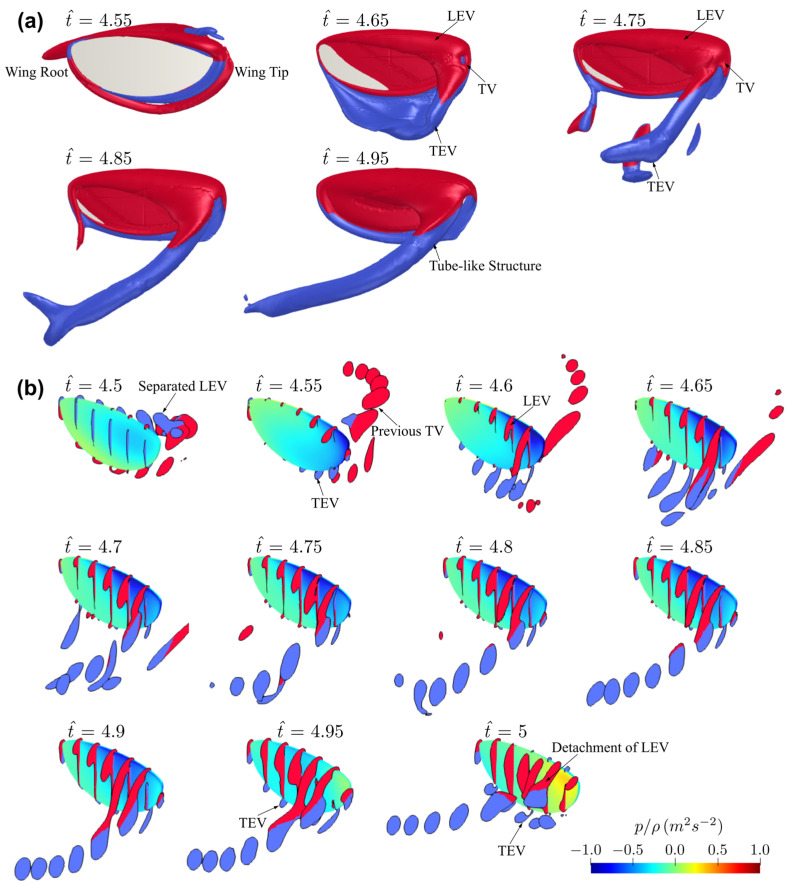
Flow evolution at different time instances for the baseline case. (**a**) Complete flow structure on the wing surface. (**b**) Flow structure at different spanwise locations. Colour map represents kinematic pressure on the wing surface. For clarity of visualisations, the wing view angle is kept constant, and thus is not reflective of the wing’s instantaneous angle of attack.

The flow evolution history for the baseline case used for the convergence study is shown in Figure 5a. The red and blue colours indicate counter-clockwise and clockwise rotating vortices with respect to the axis from wing root to wing tip. The flow structures at seven different spanwise locations between the wing root and wingtip for a range of time instances are shown in Figure 5b. In this study, the force coefficients are shown for the fifth flapping cycle where the flow has reached periodic state and initial disturbances have diminished, and the flow structures are shown for the second half of the fifth flapping cycle due to the symmetry between forward and backward half-strokes. Additionally, all average quantities, in this study, were evaluated over the fifth flapping cycle.

To provide a general view of flow structure evolution on insect wings, the flow development for the baseline wing planform is first investigated. At the beginning of the half-stroke, $\hat{t}$ = 4.5, the leading-edge vortex (LEV) starts to form on the leading-edge of the wing. The separated LEV and tip vortex (TV) from the previous half-stroke remain in the wake region near the wing surface. The wing enters the wake region at $\hat{t}$ = 4.55; at the same time, the trailing-edge vortex (TEV) starts to form at the trailing-edge of the wing. At $\hat{t}$ = 4.6–4.65, the connected LEV, TEV, and TV form a ring-like structure, and the TEV starts to detach from the wing surface, whereas the LEV remains close to the wing surface. The LEV has larger size towards the wingtip, resulting in a conical shaped vortex structure attached on the wing leading-edge. After $\hat{t}$ = 4.75, the vortex-ring develops into an elongated tube-like structure formed by the LEV and TV, which convects vorticity downstream into the wake. The flow structure remains similar until end of the half-stroke, but the LEV gradually increases in size until $\hat{t}$ = 4.95, where a new TEV starts to appear at the trailing-edge. At end of the half-stroke, $\hat{t}$ = 5, a larger TEV is evident near the trailing-edge and the LEV separates from the leading-edge and detaches from the wing surface at the outboard wing region.

### 2.4. Validation of the Numerical Set-Up

**Figure 6:**
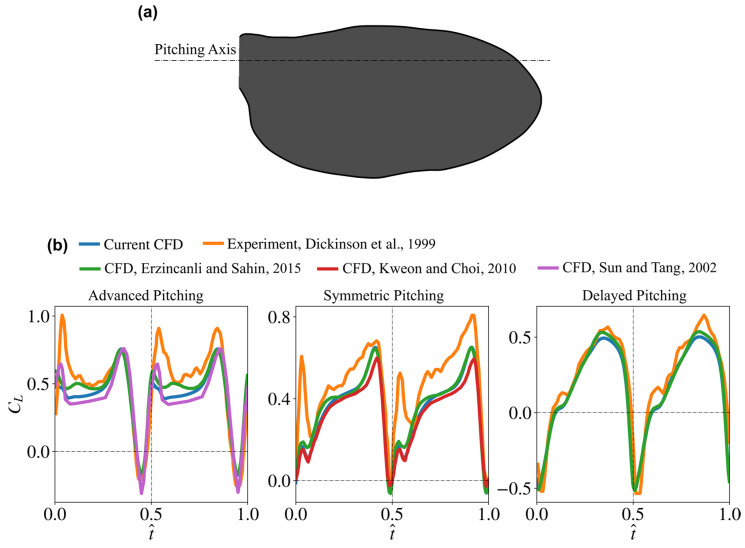
(**a**) Fruit fly wing planform shape employed within the validation study. (**b**) Comparison of the lift coefficients from the current simulation set-up against experimental (from Dickinson et al. [5]) and numerical (from Erzincanli and Sahin [44], Kweon and Choi [43], and Sun and Tang [29]) results for advanced, symmetric, and delayed pitching kinematic cases.

To provide a validation demonstration of the developed numerical solver, the lift coefficient values of the well-known experimental benchmark fruit fly case of Dickinson et al. [5] are compared against results for the same case evaluated using the current numerical set-up. Moreover, the simulation results from several CFD studies in the literature that also provided an assessment against the same fruit fly experimental case are included for comparison. Here, the wing planform shape employed for validation is a natural fruit fly wing similar to that employed by Dickinson et al. [5], and is shown in Figure 6a. The wing has an aspect ratio of 2.44, and the wing’s cross section has a thickness to mean chord ratio of 4%. The wing kinematics for the advanced, symmetric, and delayed pitching motion waveforms are all taken from the experiments of Dickinson et al. [5], and the simulations are carried out at a Reynolds number of 136. Note that all presented results from the different CFD studies employ wing planforms and kinematics consistent with that of Dickinson et al. [5]; nevertheless, in some cases, e.g., as in Kweon and Choi [43], a detailed explanation of the used planform is not explicitly provided. Figure 6b shows the comparison of the instantaneous lift coefficient values from our current set-up against all other studies. Furthermore, a comparison of the average lift coefficients (${\bar{C}}_{L}$) is presented in Table 3.

The results show that the lift coefficient values from our current simulation are in very good agreement with that reported by other studies. Only minor differences exist between the different CFD results, and these may be due to slight differences in the method of reproducing the wing planform shape and kinematics adopted by each study. Nevertheless, and more importantly, the trends and average values from our CFD simulation set-up closely follow those from the other CFD studies. Notably, all CFD results share some differences against the experimental results, which may be due to potential measurement inaccuracies or differences in the morphology/kinematics employed. That said, it is important to note that the main difference between experiments and all numerical results always occurs at the start of half-strokes, where a noticeable peak in lift is mainly seen within the advanced and symmetric pitching cases. These peaks are explained by Dickinson et al. [5] to be a result of the wake capture effect. However, such peaks have been absent from CFD simulations, and this has created one of the most well-known debates within the field of aerodynamics of insect flight where experiments show an effect which seems to be absent from numerical simulations. For more discussions about this debate, the reader is referred to [29,45]. Experimental results, however, show less evidence of such peaks for delayed pitching kinematics, and for such case, a good similarity between experiments and CFD exists. As such, overall, the qualitative and quantitative agreement between our simulations and the experimental and numerical benchmark results provides confidence in the numerical simulation set-up employed in this study.

## 3. Results and Discussion

### 3.1. Effect of Aspect Ratio

**Figure 7:**
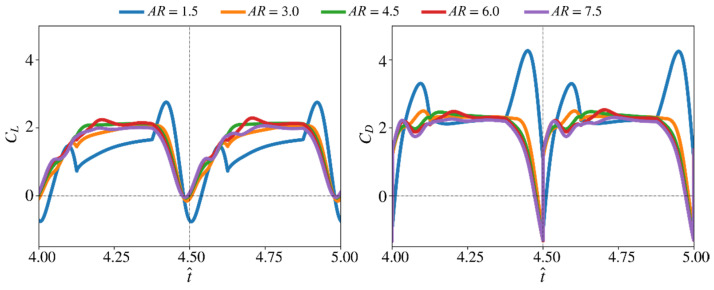
Time histories of the lift and drag coefficients for different aspect ratios. The non-dimensional radial centroid location is set to 0.5 and the wing root offset to zero.

In this section, the effect of aspect ratio on aerodynamic force production is independently studied. The other morphological parameters are set constant, within this assessment, to their nominal values: ${\hat{r}}_{1}$ is set to 0.5 and the wing root offset is set to zero, ${\hat{r}}_{R}$ = 0. Different aspect ratio values ranging between 1.5 to 7.5, with a step of 1.5, are investigated. Figure 7 shows the instantaneous lift and drag coefficients from this assessment.

The lift and drag coefficient time histories show large transient peaks for the lowest aspect ratio case (AR = 1.5), which differs from other aspect ratios whose transient force coefficient peaks near the start of a half-stroke are less noticeable and have no obvious peaks near the end of half-strokes. However, the lift and drag coefficients at mid half–stroke is lower for the AR = 1.5 case, compared with higher aspect ratio cases. In fact, higher aspect ratios show faster increase of lift coefficient at the beginning of the translational phase. After the mid half-stroke, for AR = 1.5 to 3, lift coefficient continues to increase until end of the translational phase, whereas, for aspect ratios greater than 4.5, lift and drag coefficients remain relatively constant or start to decrease.

**Figure 8:**
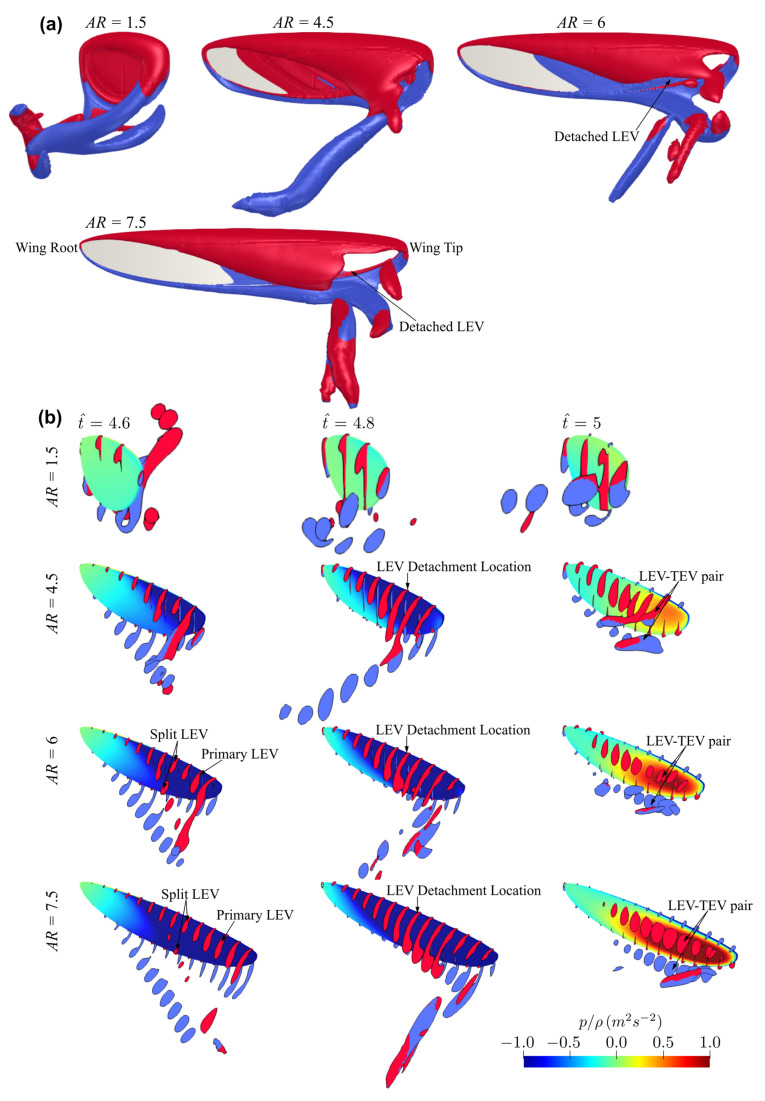
Flow structures at different time instances for the different aspect ratio cases. (**a**) Flow structure near mid-stroke ($\hat{t}$ = 4.8), (**b**) Sectional vortical structure and surface pressure field near the beginning, near the middle, and at the end of the half-stroke.

The flow structures for the different aspect ratio cases (AR = 1.5–7.5, except for AR = 3, which has been comprehensively shown in Figure 5) near the mid half-stroke (i.e., $\hat{t}$ = 4.8) is shown in Figure 8a, whereas the sectional vortical structures together with the surface pressure fields at different time instances along the half-stroke (i.e., $\hat{t}$ = 4.6, 4.8 and 5.0) are shown in Figure 8b.

Comparing the flow structures for the different aspect ratio cases, it can be seen that for the lower aspect ratio cases, the LEV is more closely attached to the wing surface, whereas the LEV becomes more distant from the wing surface as the aspect ratio increases. For low aspect ratios, the LEV gets merged with the tip vortex and shed downstream, whereas for high aspect ratios, the LEV starts to get detached from the wing surface near the wing tip. In this study, the LEV detachment location is approximately identified at the location where a local TEV is initiated. This is because when the LEV detaches from the wing surface, a TEV is formed due to flow reversal around the trailing-edge, as shown in [46,47]. Note that this criterion has been employed for identifying LEV detachment location in other studies, e.g., [20]. It is found that the detachment location moves slightly further away from the wing root as aspect ratio increases but remains at approximately $r/\bar{c}$ = 3.5–5.0 for the different aspect ratios. However, as aspect ratio increases, the LEV detachment area near the wing tip increases; it is therefore likely that the detachment of the LEV in that region leads to the relatively constant/reduced lift and drag coefficient after mid half-stroke for aspect ratio greater than 4.5. Notably, the current results agree with the experimental study of Kruyt et al. [24], which showed that the detachment of LEV or ‘stall’ location was found at $r/\bar{c}$ = 4.0. However, in their experiment, a steady revolving wing was employed, whereas in the current flapping wing case, the wing experiences cyclic formation of LEV within each half-stroke.

For aspect ratios greater than 4.5, at the end of the half-stroke, i.e., $\hat{t}$ = 5.0, an obvious high-pressure region is evident on the outboard region of the wing. The high pressure is related to the LEV-TEV pair, which can be clearly seen from Figure 8b for AR = 4.5–7.5. We note here that this high-pressure region resulting from the LEV-TEV pair in the wake of high aspect ratio wings may be significant for determining the wake capture force contribution in the subsequent half-stroke.

### 3.2. Effect of Radial Centroid Location

**Figure 9:**
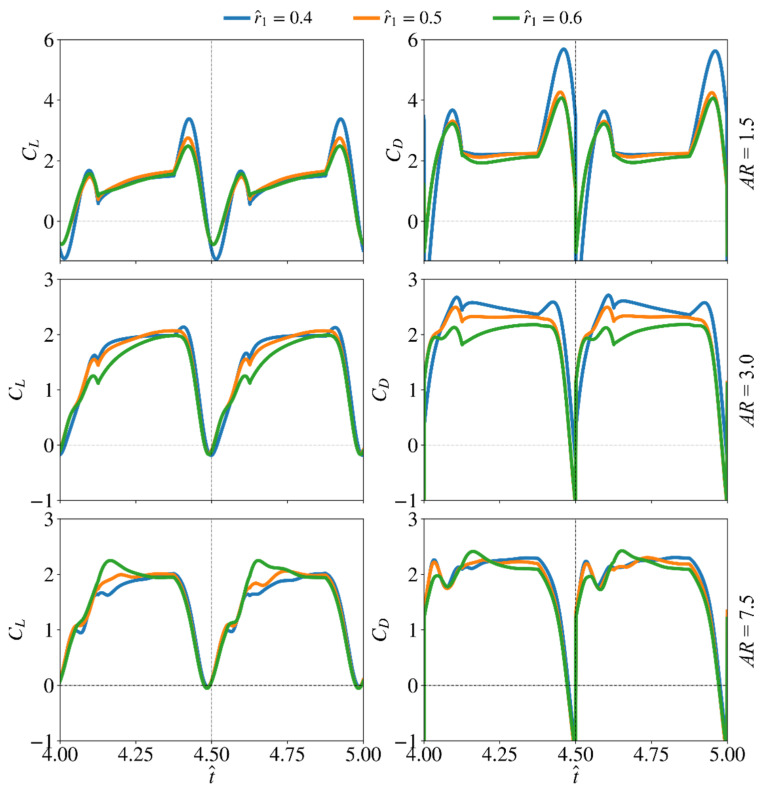
Time histories of the lift and drag coefficients for different radial centroid locations and three different aspect ratios. Wing root offset is set to zero in this demonstration.

The effect of radial centroid location on the force production and flow structure evolution is studied in this section. Here, the lowest aspect ratio (AR = 1.5), the highest aspect ratio (AR = 7.5), and a representative aspect ratio for most insect wings (AR = 3) are used to study the effect of ${\hat{r}}_{1}$. The remaining two aspect ratio cases are not included in this section to avoid excessive discussions, but their average results will be shown later in the paper. Here, the wing root offset is also set to zero, as it is most representative for typical real insects (note that the wing offset is usually a consequence of mechanical design constraints of flapping wing rigs or robotic insects, but is not common in real insects). Nonetheless, its effect will be discussed in the next section. The instantaneous values of the lift and drag coefficients are shown in Figure 9.

For the smallest aspect ratio case (AR = 1.5), changing ${\hat{r}}_{1}$ shows larger influence on the transient peaks of lift and drag coefficients near the beginning and end of a half-stroke: the peaks of the lift and drag coefficients reduce with the increase of ${\hat{r}}_{1}$, indicating reduced contribution of wing pitching rotation to force production as ${\hat{r}}_{1}$ increases. For the other aspect ratio cases (e.g., AR = 3 and 7.5), varying ${\hat{r}}_{1}$ mostly influence the coefficient values near the beginning of the half-stroke. For AR = 3, increasing ${\hat{r}}_{1}$ is found to reduce lift and drag coefficients. The reduction of lift and drag coefficients is most significant in the first part of the half-stroke, between $\hat{t}$ = 4.6–4.8. However, the lift coefficient values, for the different ${\hat{r}}_{1}$ cases, reach similar values near the end of the half-stroke. Similarly, the drag coefficient for the different ${\hat{r}}_{1}$ cases become closer towards the end of a half-stroke, but not as close as in the lift coefficient case. For AR = 7.5, higher ${\hat{r}}_{1}$ leads to higher lift coefficient values and a noticeable peak is evident for the ${\hat{r}}_{1}$ = 0.6 case at $\hat{t}$ = 4.15 and 4.65. On the other hand, the drag coefficient values for the ${\hat{r}}_{1}$ = 0.6 case are lower than the smaller ${\hat{r}}_{1}$ cases, except at $\hat{t}$ = 4.15 and 4.65.

**Figure 10:**
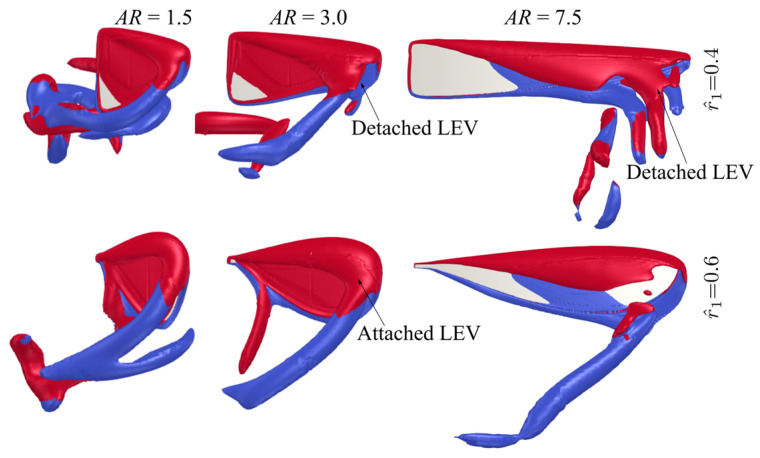
Flow structures near mid half-stroke ($\hat{t}$ = 4.8) for radial centroid location values of 0.4 and 0.6 at different aspect ratios.

**Figure 11:**
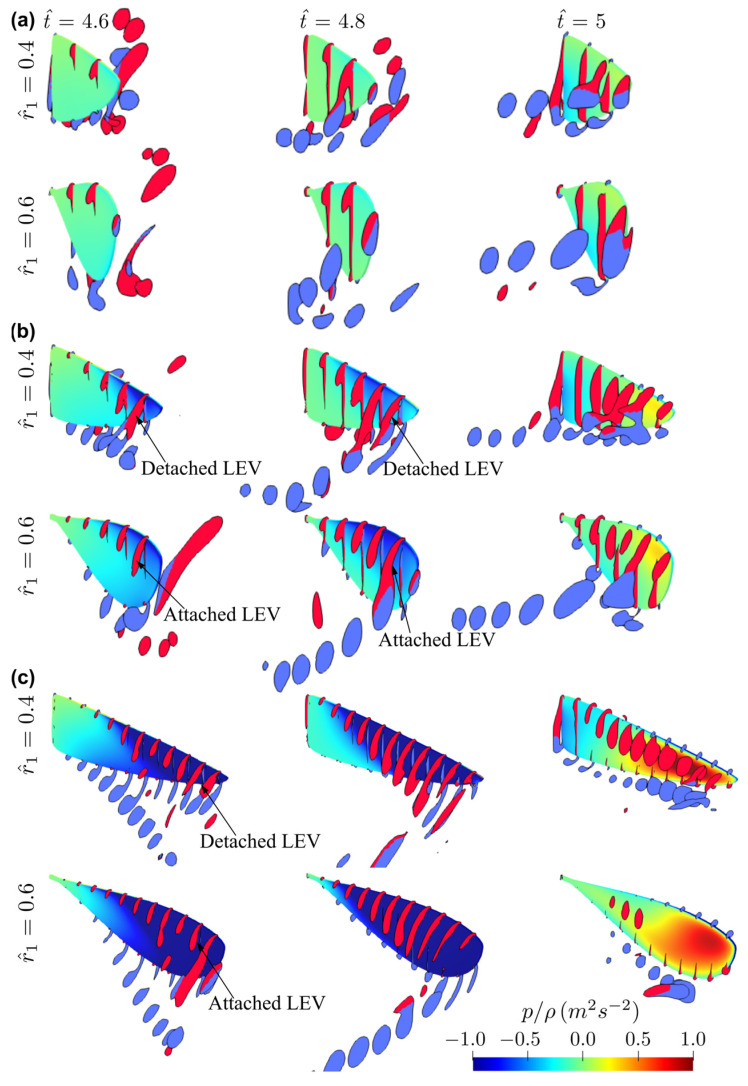
Sectional vortical structures and surface pressure fields near the beginning, near the middle, and at end of half-stroke for ${\hat{r}}_{1}$ = 0.4 and 0.6 cases. (**a**) AR = 1.5, (**b**) AR = 3, (**c**) AR = 7.5.

The flow structures for the two extreme cases with ${\hat{r}}_{1}$ = 0.4 and 0.6 at different aspect ratios (AR = 1.5, 3, and 7.5) near the mid half-stroke (i.e., $\hat{t}$ = 4.8) are shown in Figure 10. The sectional vortical structures and surface pressure distributions for the different cases at different time instances along the half-stroke ($\hat{t}$ = 4.6, 4.8, and 5.0) are shown in Figure 11. When increasing the radial centroid location, the wing chord becomes larger at the outboard section of the wing. Since the LEV typically detaches from the wing surface when the vortex boundary reaches the trailing-edge [47], this would result in different vortex formation time for wings with different ${\hat{r}}_{1}$ values. From Figure 11, it is evident that increasing ${\hat{r}}_{1}$ results in a delayed formation and thus delayed detachment of LEV near the wingtip, due to the increased local chord length at the wing outboard section. This effect is less obvious for the smallest aspect ratio wing, AR = 1.5, since for such a case, the LEV remains attached throughout the stroke. For AR = 3.0, the early formation of the LEV at lower ${\hat{r}}_{1}$ results in higher lift and drag coefficients at early half-stroke, $\hat{t}$ = 4.1–4.25 and 4.6–4.75; however, the lift coefficients finally reach similar values at later half-stroke, see Figure 9. For AR = 7.5, due to the detachment of the LEV on the outboard section of the wing, the delayed formation of the LEV at higher ${\hat{r}}_{1}$ allows the wings to sustain greater vorticity before the LEV detaches, and this leads to higher lift coefficients at the early half-stroke, as shown in Figure 9.

### 3.3. Effect of Root Offset

**Figure 12:**
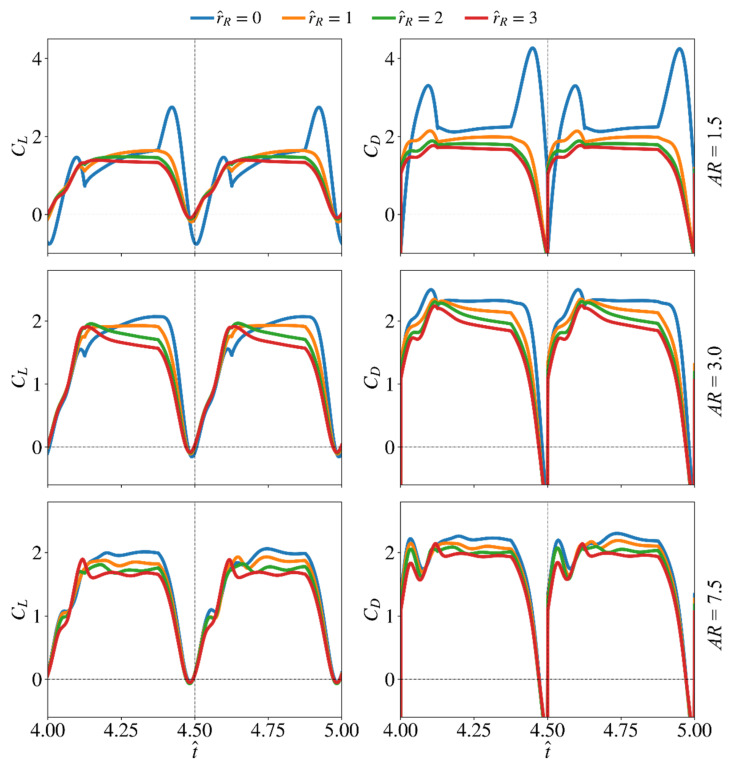
Time histories of the lift and drag coefficients for the different wing root offset cases at three aspect ratios.

The effect of root offset on the force production and flow structures is presented in this section. Here, again, the lowest aspect ratio (AR = 1.5), the highest aspect ratio (AR = 7.5), and a representative aspect ratio for most insect wings (AR = 3) are used to study the effect of ${\hat{r}}_{R}$. Only the baseline wing planform centroid location, ${\hat{r}}_{1}$ = 0.5, is considered to avoid any mixed effects—it is more convenient to change only one parameter at a time for better assessment of the outputs. The instantaneous lift and drag coefficient values for the different ${\hat{r}}_{R}$ cases at AR = 1.5, 3, and 7.5 are shown in Figure 12.

Similar to the cases of increasing ${\hat{r}}_{1}$, Figure 12 shows that for the smallest aspect ratio wing case (AR = 1.5), increasing wing root offset leads to reduced or almost diminished initial and end peaks for the lift and drag coefficients, indicating reduced contribution of the wing pitching rotation to force production as ${\hat{r}}_{R}$ increases. As evident from Figure 12, varying the wing root offset has greater influence on force production within the later phase of the half-stroke ($\hat{t}$ = 4.2–4.4 and 4.7–4.9) for all aspect ratios. In fact, when ${\hat{r}}_{R}$ increases, the lift and drag coefficients within this latter phase of the half-stroke reduce for all aspect ratio cases.

**Figure 13:**
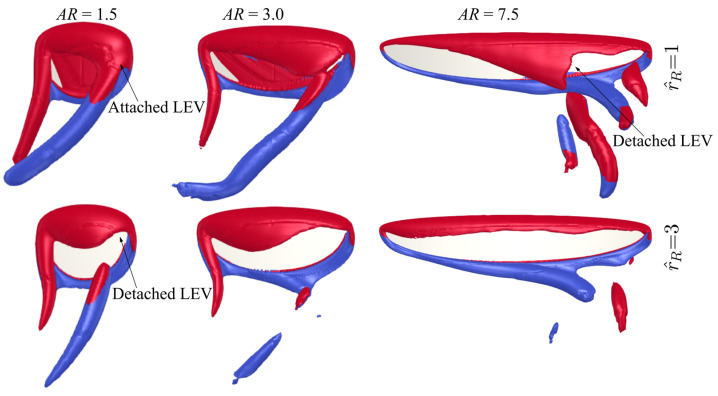
Flow structures near mid half-stroke ($\hat{t}$ = 4.8) for ${\hat{r}}_{R}$ = 1 and 3 cases at different aspect ratios.

**Figure 14:**
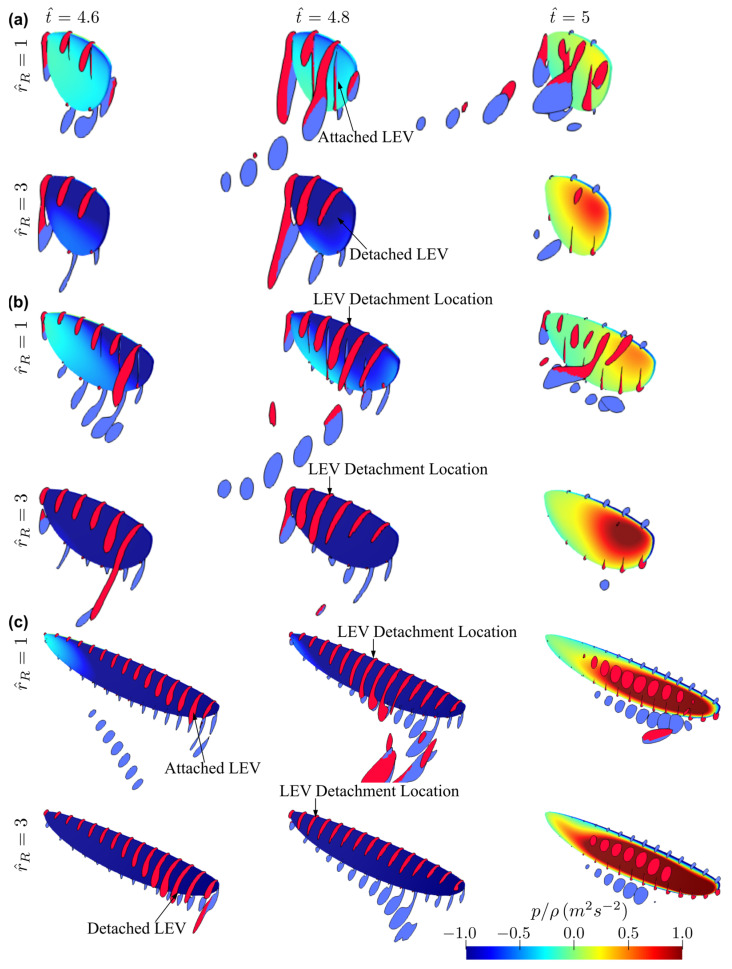
Sectional vortical structures and surface pressure fields near the beginning, near the middle, and at the end of the half-stroke for ${\hat{r}}_{R}$ = 1 and 3 cases. (**a**) AR = 1.5, (**b**) AR = 3, (**c**) AR = 7.5.

The flow structures for the two extreme cases with ${\hat{r}}_{R}$ = 1 and 3 at different aspect ratios (AR = 1.5, 3, and 7.5) near the mid half-stroke (i.e., $\hat{t}$ = 4.8) are shown in Figure 13. The sectional vortical structures and surface pressure distributions for the same cases at different time instances along the half-stroke ($\hat{t}$ = 4.6, 4.8 and 5.0) are shown in Figure 14. Note that zero-wing root offset cases can be found in Figure 5 and Figure 8. From Figure 13 and Figure 14, for all aspect ratios, increasing the wing root offset results in an increased LEV size on the inboard section of the wing near the mid half-stroke ($\hat{t}$ = 4.8); however, at the same time, the detachment location of the LEV is moved inboard, resulting in larger outboard wing area experiencing a detached LEV. For AR = 1.5, no detachment of the LEV at $\hat{t}$ = 4.8 is found for the zero-wing root offset case, as previously demonstrated in Figure 8b, but for the ${\hat{r}}_{R}$ = 3 case, detachment of LEV near the wingtip is evident as shown in Figure 14a. Similarly, for AR = 3.0, the LEV is detached on the outboard section of the wing for the ${\hat{r}}_{R}$ = 3 case, resulting in a reverse conical-shaped LEV with smaller size at the wingtip. For AR = 7.5, when ${\hat{r}}_{R}$ = 1, the LEV detachment location is moved inboard (Figure 14c), compared to the zero-wing root offset case (Figure 8b), at $\hat{t}$ = 4.8; whereas for the largest ${\hat{r}}_{R}$ = 3 case, the detachment of the LEV is almost extended to the whole wing at $\hat{t}$ = 4.8 (Figure 14c). The larger flow detachment area at higher ${\hat{r}}_{R}$ during the later half-stroke ($\hat{t}$ = 4.2–4.4 and 4.7–4.9) results in the reduced lift and drag coefficients, as shown in Figure 12.

### 3.4. Average Force Production and Aerodynamic Efficiency

#### 3.4.1. Average Coefficients for Different Aspect Ratio and Radial Centroid Locations

**Figure 15:**
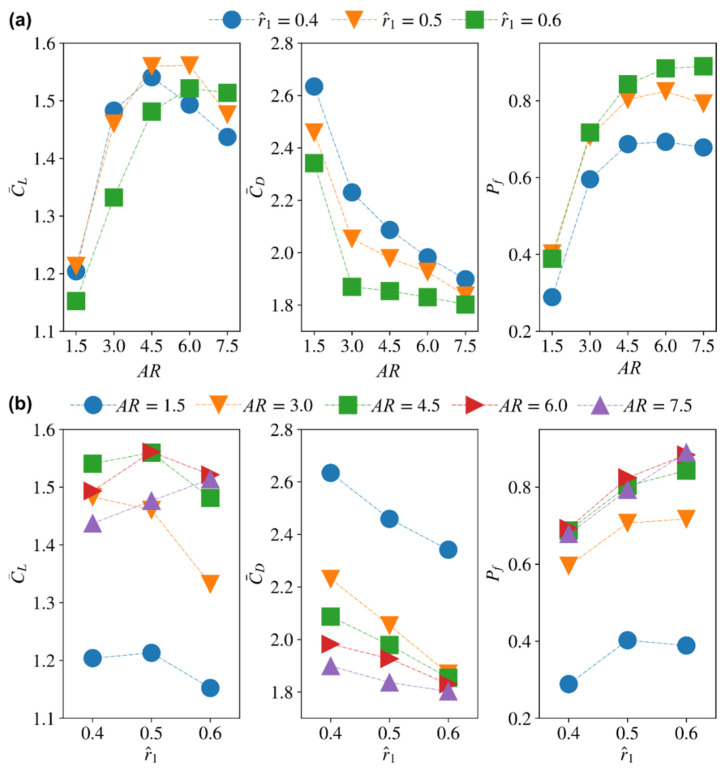
Average lift and drag coefficients as well as aerodynamic efficiency (measured in terms of the proposed power factor) variations against: (**a**) aspect ratio for different radial centroid locations, and (**b**) radial centroid locations for different aspect ratios.

To further assess the aerodynamic performance of the different wings simulated in this study, the average lift and drag coefficients, ${\bar{C}}_{L}$ and ${\bar{C}}_{D}$, and the power factor, $P_{f}$, are investigated against the different morphological parameters. Figure 15 shows the variation of the average aerodynamic characteristics versus wing aspect ratio and radial centroid location for zero-wing root offset.

From Figure 15a, with the increase of aspect ratio, the average lift coefficient first increases until it reaches the maximum value and then starts to decrease. However, the larger the radial centroid location, the peak lift coefficient gets delayed to higher aspect ratios: For ${\hat{r}}_{1}$ = 0.4, the maximum average lift coefficient occurs at AR = 4.5; for ${\hat{r}}_{1}$ = 0.5, the maximum average lift coefficient occurs at AR = 4.5–6; whereas, for ${\hat{r}}_{1}$ = 0.6, the maximum average lift coefficient occurs at AR = 6. The average drag coefficient first decreases rapidly with the increase of aspect ratio for AR = 1.5–3, but then decreases slowly at higher aspect ratios, AR = 3–7.5. This variation trend is the same for all radial centroid locations. The aerodynamic efficiency follows a similar trend as that of the average lift coefficient; however, less variation of $P_{f}$ is found for aspect ratios between 4.5 and 7.5 when compared with the corresponding variations of the average lift coefficient.

Figure 15b is another way of demonstrating the results in Figure 15a; however, plotting it in that way allows better exposure of the ${\hat{r}}_{1}\;$effect. The results show that when compared to the aspect ratio effect, ${\hat{r}}_{1}$ has relatively less influence on the lift/drag coefficient and efficiency. The variation of lift coefficient with ${\hat{r}}_{1}$ is dependent on the aspect ratio, for AR = 1.5–3, increasing ${\hat{r}}_{1}$ tends to decrease average lift coefficient; for AR = 4.5–6, the highest lift is found at intermediate ${\hat{r}}_{1}$ of 0.5; for AR = 7.5, increasing ${\hat{r}}_{1}$ tends to increase average lift coefficient. On the other hand, the average drag coefficient reduces consistently with ${\hat{r}}_{1}$ for all aspect ratio cases, which generally results in a consistently increasing trend for efficiency $P_{f}$ with the increase of ${\hat{r}}_{1}$ for most aspect ratios.

Overall, the main outcome of this assessment is that very low aspect ratios (i.e., AR = 1.5) are very inefficient aerodynamically, whereas high aspect ratios (i.e., AR ≥ 6) do not lead to a clear improvement in aerodynamic performance and are more prone to tip stall. Moreover, a higher aspect ratio wing is not favorable from a structural point of view due to the increased inertial effects whilst flapping [48]. As such it seems that the intermediate aspect ratio range (AR = 3–4.5) provides the best compromise. As such, it is not surprising to see most insects’ wing aspect ratios clustered within this range [48]. On the other hand, the current results show that the radial centroid location has less influence when compared to aspect ratio for the Reynolds number investigated (Re = 100), i.e., whilst it still has a noticeable effect on the aerodynamic characteristics, its role can be seen as more of a tuner to the performance.

#### 3.4.2. Average Coefficients for Different Aspect Ratio and Wing Root Offset

**Figure 16:**
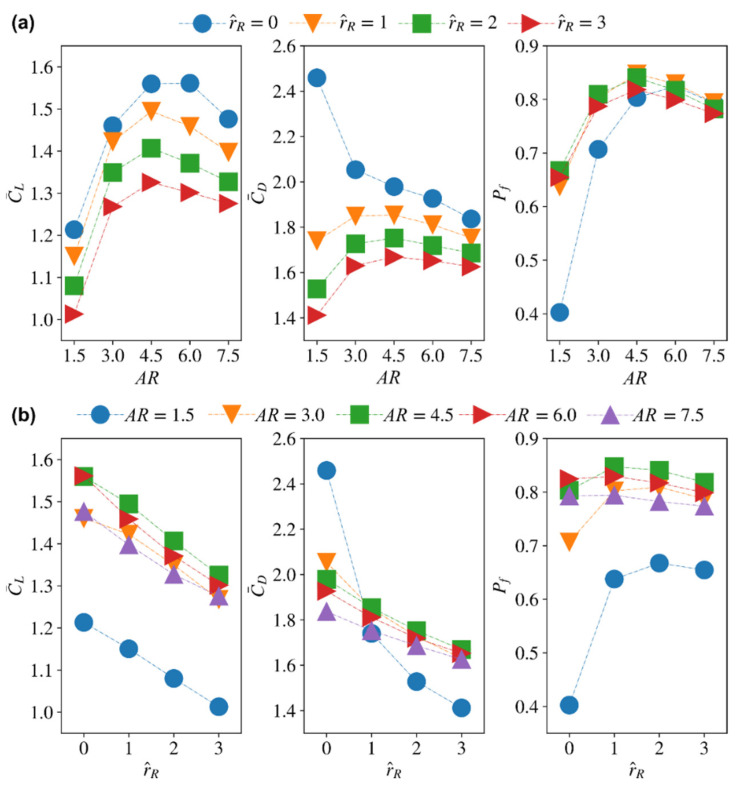
Average lift and drag coefficients as well as aerodynamic efficiency (measured in terms of the proposed power factor) variations against: (**a**) aspect ratio for different wing root offsets, and (**b**) wing root offset for different aspect ratios.

The variations of the average lift and drag coefficients, ${\bar{C}}_{L}$ and ${\bar{C}}_{D}$, and the power factor,$\;P_{f}$, against the different wing aspect ratios and root offsets for a constant radial centroid location of ${\hat{r}}_{1}$ = 0.5 are shown in Figure 16. For all root offsets, increasing the aspect ratio first increases the average lift coefficient until AR = 4.5–6, after which, the average lift coefficient starts to decrease with the increase of aspect ratio. The average drag coefficient shows similar trend and peaks at AR = 4.5 for root offsets ${\hat{r}}_{R}$ = 1–3. The only exception is the zero-wing root offset case, where the smallest aspect ratio wing, AR = 1.5, shows much higher average drag coefficients which decrease rapidly with the increase of aspect ratio. Consequently, the power factor, $P_{f},$ first increases with aspect ratio and peaks at AR = 4.5–6 for all wing root offsets, with the zero-wing root offset case showing more rapid increase at the smaller aspect ratio range (AR = 1.5–4.5).

For all aspect ratios, increasing the wing root offset decreases both the average lift and drag coefficients, Figure 16b. The resulting efficiency variation shows very little change for the aspect ratio range AR = 4.5–7.5; however, this does not mean that any wing root offset is a good selection. This is because efficiency figures should always be judged in conjunction with the lift production metrics. In this case, it is clear that increasing the wing root offset has a negative effect on average lift, and therefore when considered with the efficiency results, it becomes clear that having no or very little offset is probably the best choice. This explains why most insects have evolved with no or little offset. However, caution should be taken as insects also perform forward flights, but hovering is an important driver of their evolution given that it is a very demanding flight mode from an efficiency point of view.

The results shown here additionally highlight the need for better improvement to both experimental rigs used to measure insect wing aerodynamics as well as robotic insects as they usually possess a large offset. Such an offset can have a significant effect on the measured aerodynamic characteristics, as shown here, to the extent that results cannot be representative of a pure hovering mode anymore. Another interesting remark is that the way the wing area is distributed from the centre of rotation has a major influence on the resulting aerodynamics. This is clear when the results of changing the radial centroid location are compared to those of changing the wing root offset. Both parameters change the wing area distribution; however, their resulting effects on the aerodynamic behaviour of flapping wings are distinct.

## 4. Conclusions

In this study, the effect of wing planform morphology on the aerodynamic performance of representative insect wings is studied via numerical simulations. This is achieved by solving the incompressible Navier-Stokes equations governing the flow at a Reynolds number of 100. The wing planform shapes adopted in this study were defined using the beta-function distribution which allows for wide representation of the different insect wing shapes in nature. The planform shape parameters of aspect ratio, radial centroid location, and wing root offset were systematically investigated for typical normal hovering kinematic waveforms. The influence of the parameters on aerodynamic force coefficients, aerodynamic efficiency, and flow structure evolution were identified.

For the aspect ratios investigated, AR = 1.5–7.5, the lowest aspect ratio of 1.5 showed obvious peaks of lift coefficient near stroke reversals, which were less obvious/non-existing for higher aspect ratios. However, the drag coefficient was also high at the same incidents, resulting in significantly lower aerodynamic efficiency for AR = 1.5. Increasing aspect ratio resulted in a faster increase of lift coefficient at the beginning of translational phase, likely due to the fast growth of the LEV on the outboard wing. However, when the aspect ratio is greater than 4.5, the LEV starts to detach from the wing at approximately 3.5–5 mean chord lengths from the wing center of rotation, near the mid half-stroke, resulting in decreased lift and drag coefficients within the later part of half-stroke.

The radial centroid location is found to mainly influence the LEV formation and detachment time, due to the change in local chord length on the outboard wing: a small centroid location results in faster development/detachment of the LEV due to the small local chord length on the outboard wing, whereas a larger centroid location results in delayed development/detachment of the LEV due to the larger local chord length on the outboard wing. Consequently, for small aspect ratios, where the wing tip stall is relatively weak, smaller centroid location results in higher lift coefficient at the beginning of the half-stroke due to the faster growth of the LEV, whereas, for high aspect ratio wings, larger centroid location results in higher lift coefficient at the beginning of the half-stroke due to the delayed detachment of LEV.

Wing root offset is found to mainly influence the LEV detachment area. Increasing root offset increases the LEV detachment area on the outboard wing after mid half-stroke. This results in reduced lift and drag coefficients within the later half-stroke for all aspect ratios investigated.

Finally, it is found that the intermediate aspect ratios between 4.5–6 result in the highest average lift coefficient for all radial centroid location and root offset cases. The optimum aerodynamic efficiency also lies within the same range for most cases; however, the optimum aspect ratio for efficiency is delayed to 7.5 for high centroid location cases. Increasing centroid location results in relatively small variations in lift coefficient, however, aerodynamic efficiency increases with the increase of centroid location. Increasing root offset is found to decrease both average lift and drag coefficients, but results in small variation in aerodynamic efficiency for high aspect ratio wings.

Overall, our results show that very low aspect ratio of 1.5 is very inefficient aerodynamically, whereas high aspect ratio greater than 6 do not lead to a significantly higher aerodynamic performance and in fact are prone to tip stall. As such it seems that the intermediate aspect ratio range (AR = 3–6) provides the best compromise. On the other hand, radial centroid location and root offset show relatively less influence on aerodynamic performance when compared to aspect ratio; however, they act as tuners to the aerodynamic performance of insect wings.

## Figures and Tables

**Table 1:** Morphological parameters describing the wing planforms considered for investigation.

Morphological Parameter	Values
AR	1.5, 3, 4.5, 6, 7.5
${\hat{r}}_{1}$	0.4, 0.5, 0.6
${\hat{r}}_{R}$	0, 1, 2, 3

**Table 2:** Average lift and drag coefficients for the convergence study.

Mesh Cells, Time Step	${\bar{C}}_{L}$	${\bar{C}}_{D}$
3 million cells, Δt = 1 × 10^−3^	1.460	2.046
6 million cells, Δt = 1 × 10^−3^	1.471	2.052
3 million cells, Δt = 5 × 10^−4^	1.460	2.046
6 million cells, Δt = 5 × 10^−4^	1.470	2.052

**Table 3:** Comparison of average lift coefficients from studies used for validation.

Kinematic Case	CFD, Current	Exp., Dickinson et al. [5]	CFD, Erzincanli and Sahin [44]	CFD, Kweon and Choi [43]	CFD, Sun and Tang [29]
Advanced	0.405	0.491	0.438	-	0.387
Symmetric	0.340	0.473	0.360	0.323	-
Delayed	0.170	0.237	0.183	-	-

## Data Availability

The data presented in this study are available on request from the corresponding author.
